# Perceptions of underlying practice hierarchies: Who is managing my care?

**DOI:** 10.1186/s12913-021-06931-1

**Published:** 2021-09-03

**Authors:** Tara N Officer, Karen McBride-Henry

**Affiliations:** 1 Health Services Research Centre, Victoria University of Wellington, PO Box 600, Pipitea Campus, Wellington, New Zealand; 2 School of Nursing, Midwifery, and Health Practice, Victoria University of Wellington, PO Box 600, Newtown Campus, Wellington, New Zealand

**Keywords:** Nurse practitioner, Pharmacist prescriber, Patient-centred care, Hierarchies of practice

## Abstract

**Background:**

The introduction of new health professional roles, such as that of the nurse practitioner and pharmacist prescriber in primary health care can lead to changes in health service delivery. Consumers, having used these roles, often report high satisfaction. However, there is limited knowledge of how these individuals position nurse practitioner and pharmacist prescriber roles within existing practice structures.

**Methods:**

Semi-structured interviews were conducted with 21 individuals receiving services from these practitioners in New Zealand primary health care. Interviews were recorded and transcribed verbatim for thematic analysis.

**Results:**

Participant views reflect established practice hierarchies, placing advanced practitioners ‘below’ general practitioners. Participants are unable to articulate what it was about these practitioners that meant they operated at lower tiers and often considered practitioners to act as ‘their doctor’. They also highlight structural barriers impairing the ability of these providers to operate within their full scope of practice.

**Conclusions:**

While seeing value in the services they receive, consumers are often unable to position nurse practitioner and pharmacist prescriber roles within health system contexts or to articulate how they value their practitioner’s skills. Embedded structural barriers may be more visible to consumers than their interactions with the health system suggest. This may influence peoples’ ability to receive intended or optimal health services. Consumer ‘health professional literacy’ around the functions of distinct health practitioners should be supported so that they may make informed service provision choices.

**Supplementary Information:**

The online version contains supplementary material available at 10.1186/s12913-021-06931-1.

## Background

Embedding advanced practitioner roles, including those of nurse practitioners (NPs) and pharmacist prescribers (PPs), into general practice is increasingly common globally. Introducing these roles into care teams creates a change in skill mix, and presents opportunities and challenges to role substitution, delegation, and innovation [1–3]. These changes are often considered alongside the concept of role ‘encroachment’, where working as an advanced practitioner is perceived as leading to role-scope movement, in the case of NPs, across a continuum of skill from nursing towards medicine [4]. Concurrently, debate around the intended roles of advanced practitioners, including in government position papers, academic research, and within unions, often means that advanced practitioners, particularly NPs, are classified as ‘mid-level’ health professionals. Such discussions may in turn influence the way patients and others view advanced practitioners [5, 6]. Understanding the influence these hierarchies have on how individuals view services they receive is important when planning patient-centred service provision.

The consumer^1^ is the focus and recipient of care. Movements towards multidisciplinary care teams are likely to result in changes to how these individuals receive care, yet, there is little research on how they view this change. Consumers, perceiving NPs or PPs as supplying inferior services, or who are unaware of the existence of these practitioners, may choose not to use their services. In studying the views of general practitioners (GPs) on NPs, Wilson et al. [7] suggested that GPs believe patients, particularly older patients, are resistant to using NP services. In their study, GPs suggested that patients would believe doctors were the health professional most able to make appropriate diagnoses [7]. Van Soeren et al. [8], in their study of NPs in Ontario primary health care, noted that lack of public understanding regarding the NP role discouraged the embedding of NPs in the workplace. Similarly, McCann and colleagues [9], while considering patient perspectives on pharmacist prescribing in Northern Ireland, reported a lack of patient awareness of the relative capabilities of medical doctors and PPs. In their United Kingdom study, Hobson et al. [10] remarked that poor patient awareness of pharmacist training and knowledge altered patient confidence in consulting a PP. This lack of confidence may influence the range of services advanced practitioners offer and change the acceptance of these services [11].

In the Netherlands, Laurant et al. [12] explored patient preference and satisfaction between seeing a NP or GP. Findings of their questionnaire (50 % response rate) suggest a general preference for GP services for traditional ‘medical’ aspects of care, such as receiving treatment and discussing physical complaints. However, patients also showed high general satisfaction with both NPs and GPs, although slightly higher satisfaction with NP care. In 2018, a Cochrane systematic review on the impact of nurses substituting for primary care doctors [13] suggested that patients are at least, if not more, satisfied with nurse-led care than with doctor-led care. In comparison, in their systematic review of stakeholder views and experiences with pharmacist prescribing, Jebara and colleagues [14] report general public and patient support for the PP role both pre- and post-implementation. Similarly, Famiyeh and McCarthy’s 2017 scoping review [15] documenting patient views of pharmacist prescribing, found public support for pharmacist prescribing in a range of situations, including repeat prescribing. The authors acknowledged that patient and public support for these roles was influenced by having a positive established relationship with the pharmacist.

### Study and New Zealand professional context

New Zealand-based advanced practitioners are independent prescribers, that is, they can prescribe autonomously within the limits of their governing regulations and practice scopes [16, 17]. Unlike NPs, whose prescriptive authority is limited only by practice scope [18], PPs may prescribe only subsets of registered medicines based on health profession-specific regulations. In addition, PPs must work within collaborative health team environments [19]; that is, within multi-disciplinary teams where consumers are the “focus and beneficiary of the collaboration” ([20], para. 1). PPs may not act as primary diagnosticians [19], meaning that consumers will also be under the care of other clinicians; when registering they must also submit a practice plan outlining their area of practice. In contrast, NPs can work independently and as part of health care teams. As of 2019, over 350 NPs are practising in New Zealand, with over 40 % practising in primary or community care [21]. In 2020, 34 individuals held pharmacist prescriber annual practising certificates, and as of August 2016, half worked in primary care or between primary and secondary care [22].

No formal standardised referral pathway exists for accessing NP or PP services. Indeed, individuals may have a NP acting as their first point of contact within primary care. Funding for the roles of NPs and PPs also differs across the country. Some primary care practices are NP-run, and others employ NPs or PPs, either directly, or via funding from secondary care. These differences influence how the role operates in practice and whether they are considered part of the primary care team, or as a separate practitioner operating on shared real estate. As such, the implementation of NP and PP roles is complex, a full characterisation of this complexity can be found elsewhere [11].

In New Zealand, several distinct opinions dominated discussions around the development of advanced practitioner roles. For example, in 2000, Editors of the New Zealand Medical Journal speculated that independent nurse prescribing would “inevitably increase fragmentation of care and stifle inter-professional cooperation” ([23], p. 411). Moreover, Moller and Begg [24] suggested that the nurse training and knowledge base was insufficient to ensure appropriate diagnostic skills for independent nurse prescribing. More recently, our work suggests that consumers feel confident in themselves and their clinicians as a result of their NP/ PP’s intervention [25]. Yet, there is a dearth of research on NP and PP roles that considers where consumers place the advanced practitioner in overall hierarchies of practice. While discussion has often centred on patient satisfaction, and to some extent experience, underlying public recognition of these roles has received scant attention. Importantly, no research has explored this phenomenon in individuals receiving services from NPs and PPs in New Zealand. This paper lays out consumer perceptions of where the positions that NPs and PPs hold sit within traditional practice settings in New Zealand primary health care.

## Methods

### Data collection

**Fig. 1:**
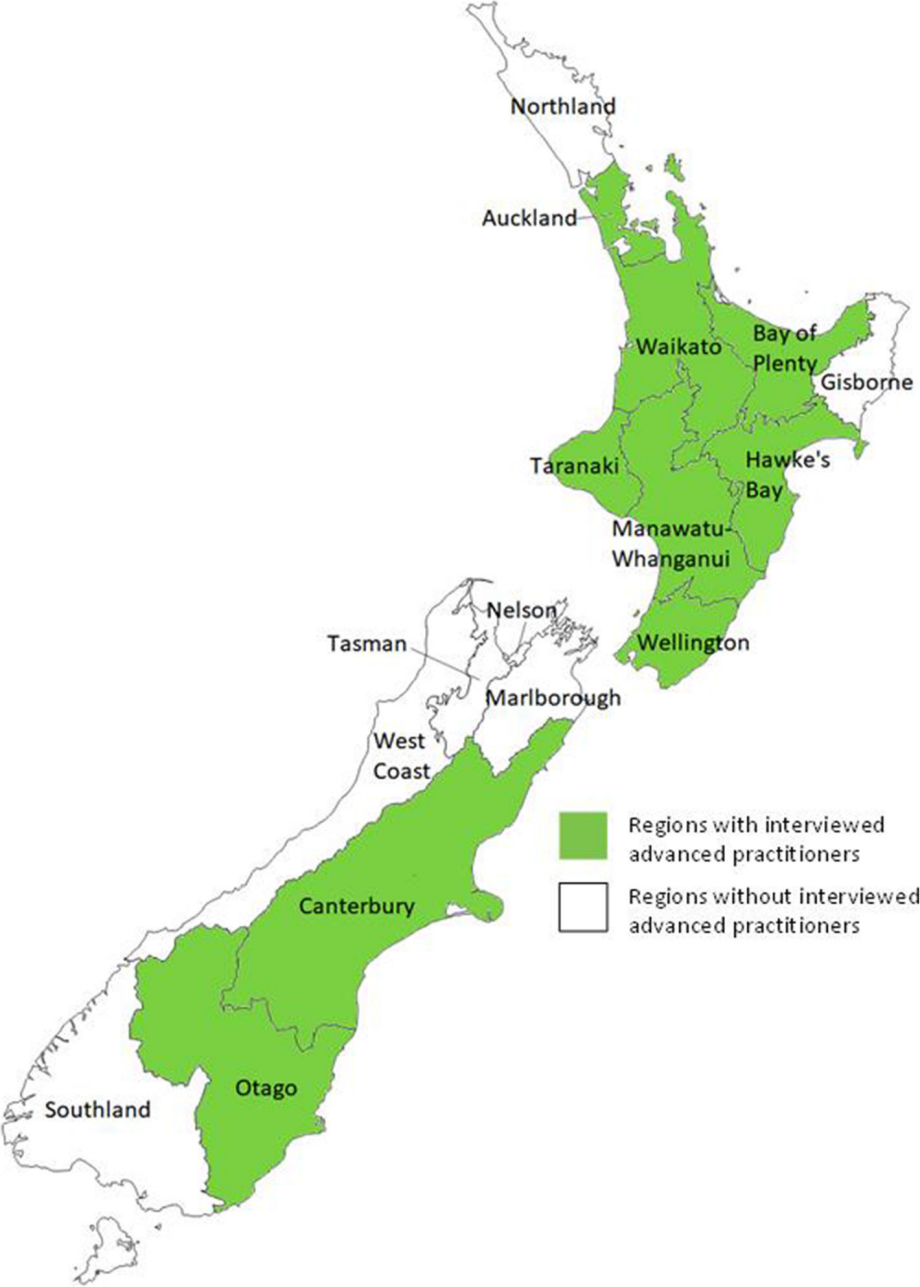
Location of participating NPs and PPs by region. Author’s creation reproduced with permission Author [11]. Map created by the author using Microsoft Paint

The primary investigator, a registered pharmacist, conducted semi-structured interviews in person with consumers (patient or carer) of NP/ PP services in mid-late 2016. These individuals received services for a range of acute and chronic conditions and had different health and access relationships with their primary care practice, some having enrolled with their practice within the past year, and others having multigenerational interactions. NPs/ PPs provided a variety of services to consumers, in the case of PPs, this was often following referral from another provider Practitioners operated in the regions in green in Fig. 1, below. People were eligible to participate in this study if they had received NP/ PP services at least once in the past year, were currently receiving services from their NP/ PP, and were not acutely ill or facing extenuating circumstances. Where individuals were under 18 years of age, or otherwise unable to consent, a carer was instead invited to participate in the research.

Advanced practitioners assisted in selecting participants, they generated blinded lists of people meeting the inclusion criteria from their daily appointment calendar. NPs/ PPs then randomly selected one person they treated each day for each of four consecutive days, explained the research, and provided them with an information sheet, consent form, and prepaid envelope to contact the research team. Where the NP/ PP managed small populations, treated people unable to consent (children or older adults), or had recently been employed by their primary care practice, then they approached fewer people. When consumers made contact, the primary investigator re-explained the research topic and agreed on a time and place to conduct an interview.

**Fig. 2:**
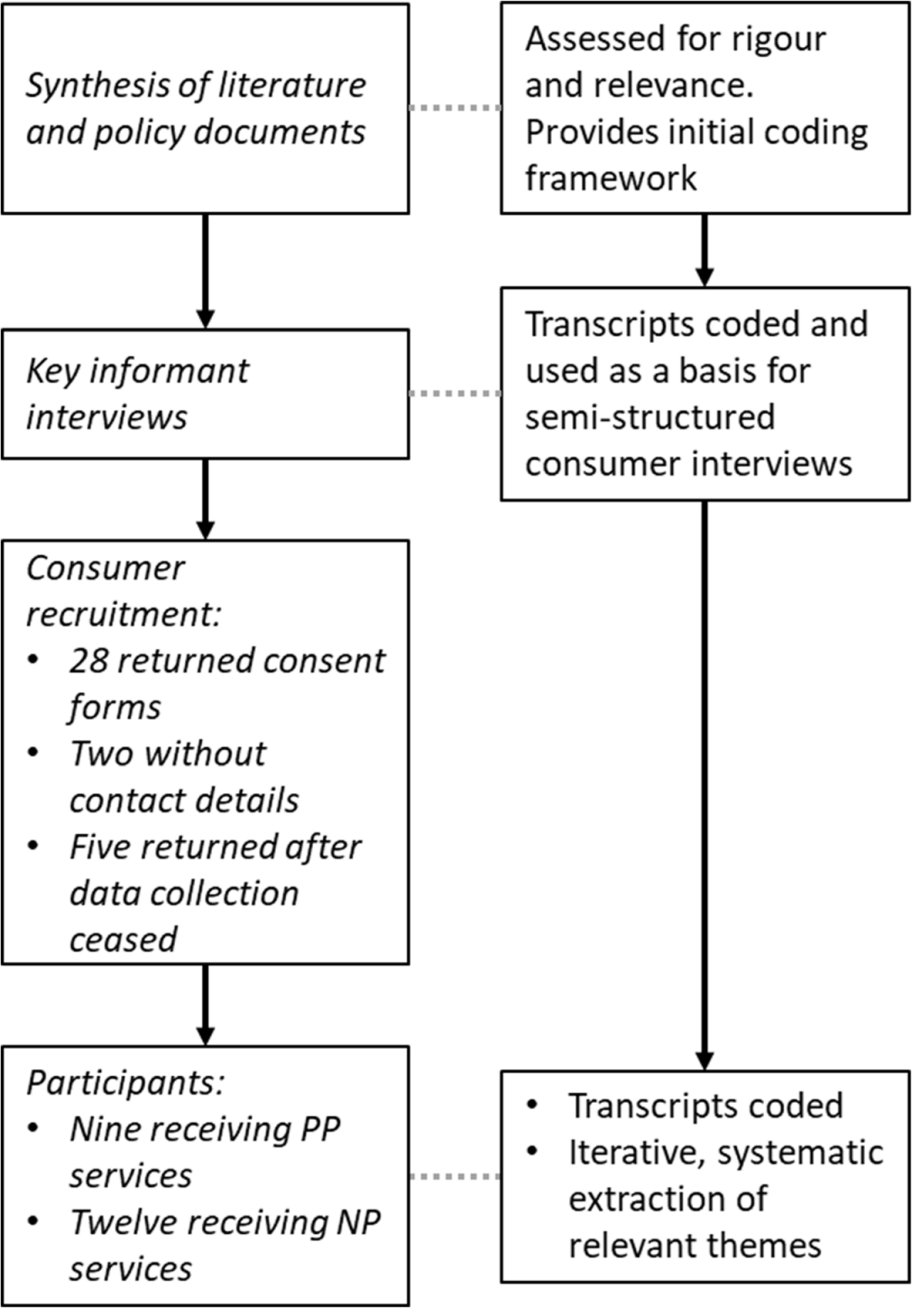
Study flow chart

Twenty-one individuals participated, nine received PP services and twelve received NP services; all had previous experience consulting with a GP (see Fig. 2). Of these, 19 participants received care directly from their advanced practitioner, the remaining three acted as carers. All advanced practitioners had considerable experience in their professional fields, although some were new to their current workplace.

All participants consented in writing to participate in an interview and have it audio-recorded and transcribed. Participants were aware that the primary investigator was a pharmacist, and that they could withdraw from the study, not answer questions, or stop the interview/ recording at any time. Interviews occurred at a mutually convenient location and lasted between ten and forty-five minutes (averaging 22 min), concluding when the participant stated they had nothing additional to discuss. Participants were advised that they could bring a support person to the interview; nobody took up this offer.

Interviews were semi-structured and began with an explanation of what the research involved. Discussions were guided by a schedule developed following review of extant literature, analysis of earlier key informant interviews (*N* = 23), and discussion within the research team. The schedule (see additional file) focused on three areas:

1. relationships with advanced practitioners;
2. factors affecting NP/ PP role introduction; and.
3. the impact of advanced practitioner roles on service provision.

Participants had the opportunity to explain and clarify their thinking during the interview process. This meant that the content of the interviews was led by the interviewees as they elaborated on their experiences and thoughts around their NP/ PP’s role.

### Data analysis

A third party working under a confidentiality agreement transcribed interviews verbatim. The primary investigator then read and re-read transcripts after checking them for accuracy against the original recording. Summaries of interviews were created, informed by fieldnotes made during data collection and further elaborated on during data analysis. Interviews were completed when similarities in stories emerged, indicating that theoretical sampling had been successful [26]. Participants who requested a copy of their transcript or interview summary were then provided with these and asked to check them for accuracy and completeness as part of ensuring that the interpretation of findings represented participant’s perspectives [27]. Thirteen participants requested copies or summaries of their transcripts.

Using NVivo 11 Pro, data was coded against interview topics and comparative analysis processes applied so that emergent themes could be tested in analysis of subsequent interviews. Informed by a review of relevant literature, and initial analysis of interviews using a realist evaluation methodology [11], data for this analysis were coded descriptively by the primary investigator and initial themes identified and refined with the research team, in keeping with processes described by Braun and Clarke [28]. Such a process ensures that common threads were identified across and between interviews [29]. To ensure findings were not influenced by earlier work, the team used clean uncoded transcripts. Themes were further interrogated and grouped to ensure that as categories emerged, they were identified and grounded in the data.

## Results

Consumers described hierarchies in which advanced practitioners operate based on their perception of their NP/ PP’s abilities. They informally benchmarked these practitioners against medical doctors (when describing skills and training) and positioned them alongside traditional nursing and pharmacy roles (when describing the value of the role). Finally, participants explained how embedded structural hierarchies exhibit themselves through care interactions. The findings highlight that such hierarchies between health professionals and barriers in service delivery influenced their views of these providers.

### “How I perceive it”

Most study participants had experience with only one NP/ PP, that is, they had only limited experience with advanced practitioners. In the case of PPs, often this experience came about because of first referrals by GPs or other health providers. This resulted in participants ‘placing’ their NP/ PP in the context of roles with which they were more familiar. Participants often perceived that their NPs and PPs practise in the interstices between traditional nursing or pharmacy roles and medicine. They, therefore, sought assistance from their advanced practitioner in situations that they found less pressing. As one participant describes:

*She is the pharmacist who instead of going to the doctor, she’s a step before that, is how I perceive it in terms of walking you through your medications and adjusting those accordingly… It’s possibly saving doctors’ appointments… The fact that she can actually write out prescriptions… That’s how I see it, somewhere between the nurse and the doctor. (P4)*

In turn, this perceived scope meant that participants considered their NP/ PP as operating either as a substitute or complement to their GP. As a substitute, these practitioners were understood to have a role to replace GPs in situations of workforce shortages. As a complement, NPs and PPs could “take some pressure off a doctor so they can… focus on more people, and then the pharmacist or the very competent nurse [can]… offer an even wider service” (P12). P2 described this well when stating that their NP is a substitute to their doctor, but not necessarily a substitute who provides an identical quality service:

*Her role is backup for the doctor. She is a substitute doctor, in a way. I don’t see her as the absolute frontline because I suspect that the doctor’s got a wider experience when it comes to really fine diagnosis… I see her as someone you can go to for most things, and if they say the doctor’s busy, we’ll give you to [the NP], then that’s just fine. (P2)*

The idea that a NP was a competent health care professional but not at the ‘level’ of a GP was explored by some participants during their interviews. One participant offered the following description of their perception of the difference and embedded hierarchy between a NP and a GP:

*She was qualified to do your scripts, and that if you had something seriously wrong, then you could be seen by the doctor if you needed to be, but on your day-to-day general maintenance of what things you’re on, you didn’t need to see anybody higher up. (P16)*

In addition, to the perception that NPs were at a different ‘level’ or ranked as less skilled than a GP, participants also considered the role of the PP in care provision and mused about ‘where’ they fitted into the health care system. Several participants described being referred to their PP, often in the first instance by their GP. Participants explained that during their first appointment, they frequently lacked knowledge of why a PP was to now treat them or what the PP’s skills were. The same situations also often held for individuals receiving NP services. Participant 17 highlighted this issue when they discussed a conversation they overheard about their NP:

*They [other patients] were asking when do we see the doctor? They didn’t understand that they didn’t have to see the doctor, like she could do everything. (P17)*

Participants often did not recognise the limits of a NP/ PP scope. They were exposed to these limits by discussing either new medical diagnoses (with PPs), or through asking their NP to assist with services outside their scope, such as drivers licence renewals (a service NPs have only been legislated to provide since 2018). Frequently, while discussing these boundaries of practice, participants described the practitioner’s skill with reference to the practice of medicine and the place the practitioners held within the hierarchy.

### Benchmarking against medical roles

Participants commented on the abilities of their advanced practitioners. They often associated being highly skilled with having qualifications in the field of medicine and described their advanced practitioner as ‘being’ their doctor, or ‘like’ their doctor. In the following quote, Participant 11 described her NP as being her children’s doctor, suggesting that she has “full confidence” in her. In turn, she suggested that the title ‘nurse practitioner’ may denote less skill, and therefore, hold less power, than the title ‘doctor’:

*I actually have full confidence in her, that if she’s not 100 % sure she is going to check with someone more senior, title-wise. But as far as I’m concerned, she does the job of a doctor. She’s my kids’ doctor and that’s what I call her, I don’t call her a nurse practitioner. (P11)*

This sentiment was also echoed by another participant as meaning that their advanced practitioner was not as skilled as a medical doctor; however, the participant recognised that a NP would refer to a more knowledgeable GP if they were not sure about a diagnosis or treatment, which made participants feel comfortable accessing the care provided by a NP/ PP.

*Okay, they’re probably not as clued up as the doctor is, but if they’ve got a problem they’ll talk to the doctor. If I’ve got a problem that they can’t work out, they will get back to me, by talking to somebody that’s clued up. (P5)*

While research participants were generally unaware of the additional training required to become an advanced practitioner, some participants associated this additional training with working towards a medical qualification:

*It’s the level that [the NP] works at. She’s not just a nurse. When I’ve been in hospital and stuff like that, the nurses are busy doing their piece but obviously [my NP] is higher. Whether she’s training to be a doctor I don’t know, but I have every confidence in her. (P10)*

In turn, this meant that advanced practitioners were described as operating as trainees. Participants struggled to identify the NP/ PP as an independent entirely different type of health care practitioner, constantly returning to what they had known previously. One participant offered the following description:

*To me, it’s like she’s like a trainee doctor. That’s how I see it. She’s not a nurse and she’s not a doctor; she’s a kind of in-between that can do prescriptions for kids, but not for adults. (P11)*.

In contrast, some participants also suggested that they would prefer to seek care from medical doctors but recognised that some NPs/ PPs could offer more specific services that met their needs appropriately. The understanding that a GP was the principal health care provider was evident in participant accounts, as the following example shows:

*I do not see why it has to be only one particular type or class of people within an occupation who deal with it… Okay, you have a doctor, and everybody wants to see the doctor, but I’m sure there’s very capable nurses or pharmacists who can deal with a lot of the same things that doctors do, and in some cases, I feel they’d probably even deal with it better. Because they become specialised, perhaps in a smaller field, as opposed to a doctor or general practitioner who has to know so many different areas and I’m sure you can’t master all of them. (P12)*

### Positioning within traditional nursing and pharmacy practice

In many instances, participants suggested that while their advanced practitioner may have explained their role to them, what made them realise the benefit of their services was building a relationship with their NP/ PP. Participants described the value of these services through comparison with traditional nursing or pharmacy roles. For example, while recognising that PPs could prescribe, participants often did not recognise differences in practice scope and instead suggested that a traditional community pharmacist role also included prescribing. Participant 12 described this as follows:

*As a member of Joe Public, I have no real understanding of the degree of knowledge that a pharmacist or a chemist in a chemist shop has… Whereas when you have a chance with someone like [my PP], I don’t know whether [she] is a typical pharmacist or whether she is more advanced in terms of knowledge… I’m impressed with her knowledge, but I have no idea of what a chemist in a pharmacy shop or whatever, of what their competency is. (P12)*

When considering intra-professional hierarchies, participants struggled to recognise differences between advanced practice and ‘base’ nursing and pharmacy roles, highlighting their confusion about where roles began and ended. When considering the NP role, participants explained the difference between this role and that of a nurse as follows:

*I’ve dealt with about four or five different nurses there… All provide good service but I suppose because of [my NP]’s level she can answer the difficult questions, where some nurses will say “oh you’re best to talk to the doctor”… When I was in there [hospital], there’s obviously different levels of nurses… but until I worked it out… that she was a nurse practitioner, I didn’t realise she wasn’t a doctor. (P10)*

Some participants further suggested that NPs acted as nursing leaders within the general practice, recognising their skills as being split between clinical practice and building the capability of other nursing staff through leadership. In one case, P20, having awareness of the leadership component of their NP’s role, stated the following:

*Often the nurse practitioners, because they’re the kind of senior nurse in the practice they also take on a lot of nursing leadership for the other registered nurses… I think if they’re having to carry their quite heavy caseloads and their own autonomous workstream and then carrying maybe nurse leadership roles for the rest of the practice, I think that can be very difficult. (P20)*

### Embedded structural barriers and hierarchies

The hierarchies that participants discerned were further perpetuated by visible structural barriers present within a practice and in the health system that affected access to timely health care. The following example, offered by a participant, highlighted a system that privileged GPs. In this case, the ‘system’ did not allow for ‘other’ health practitioners to be notified of test results, directly impacting the speed at which health concerns could be addressed. In this case, discharge information sent to a GP meant there was inadequate follow-up care:

*Even though [my NP] had done all the work of getting me into there, everything came back to [the GP]. What I got from the hospital said that they wanted my warfarin reviewed… It’s now three weeks later… I had a whole list of things to ask [my NP] yesterday and one of them was had they got the letter?… They had actually sent it to [the GP]…, so she hadn’t seen it. I don’t know whether [the GP] had seen it and ignored it or not, so until yesterday there had been no review of the warfarin. (P8)*

Another participant, noting the same barrier, highlighted privacy issues that could be exacerbated by the established hierarchy preventing an individual’s chosen health care professional accessing necessary information:

*The letters always get addressed back to the doctors… Where it says doctor information at the hospital when you’re filling in your forms I put nurse practitioner information but the letters that came back in response were always to the doctor… the system isn’t ready to accept the change maybe… You do read those letters and you’re like that’s not who I want that information to go to. What are they going to do with that? I want that to go to the person who’s managing my care. (P20)*

In addition to structural barriers, interviewees noted hierarchies they had seen in how health professionals interact with each other. In-house ‘behind the scenes’ politics between health professionals, translated into interactions that consumers saw. In some cases, participants felt their advanced practitioners were not accepted by other health professionals. One carer highlighted the frustrating nature of health profession hierarchies, noting impacts on the care they could provide:

*They don’t recognise and they don’t understand what her [NP] role is… There is a little bit of ‘well I work in the DHB, so, therefore, I’m better than you’… It’s just ridiculous, absolutely ridiculous, but it is incredibly frustrating at the same time because it hinders the care [provided]. (P19)*

On the other hand, in combination with perceived hierarchies, participants also recognised these structural hierarchies to mean that their NP/ PP would have an alternative health professional (the GP) to turn to if the need arose:

*They’re short of doctors up here at the moment anyway… I suppose it’s good in one way there, or excellent…because if necessary, she’ll [PP] call the doctor in. It’s not a bad thing, it’s probably a good thing, because normally it’s bugger the doctor until you’re really bad. (P13)*

## Discussion

The present paper lays out views from one important sector of health care provision that is often underrepresented, the consumer. In New Zealand and overseas, individuals generally express positive views of their experience using advanced practitioner services [10, 30–38]. Yet, our study suggests that participants struggled to ‘position’ advance practitioners as a health care delivery cog, and described them as operating ‘below’ GPs but ‘above’ traditional nursing or pharmacy roles. Such findings are widely supported by earlier pharmacy and nursing research [9, 10, 15, 39–41] that suggest consumers have limited awareness of the value or contribution of advanced nursing and pharmacy roles or the training that underpins their practice. This lack of awareness may limit the further development of individual NP and PP roles [9, 41], which is an issue in countries such as New Zealand where these roles are still relatively new. It is noteworthy that, unlike earlier work, participants in the present study did not suggest that this limitation presented a barrier to their decision to use advanced practitioner services.

Study participants indicated that they were not always able to perceive differences between NPs/ PPs and medical doctors. Based on this finding, caution is potentially needed when interpreting end-user satisfaction and quality of experience with advanced practitioner services [10, 30–38]. Where people do not recognise a difference between provider types, they are unlikely to ‘measure’ the quality of the services they receive accurately. In turn, this may limit the value of any inferences made from research on consumer satisfaction.

This study demonstrates that consumers continue to position NPs and PPs as either ‘mini doctors’ or aspiring to become doctors, despite (in the case of the NP) their introduction into the New Zealand context almost 20 years ago. Descriptions such as this or as “mid-level health providers”, do not define skills or competencies [5, 6], but can be harmful to how roles are considered (whether they are threats [42, 43]) and to how other establishing roles, such as the clinical nurse specialist are viewed [44]. As participants suggest, consumers often position their advanced practitioner as being ‘like their doctor’, indicating, to some extent, the perceived value of these practitioners. Where consumers are treated by multiple different providers or where structural barriers exist (such as discharging providers failing to recognise the role of new health professionals or automated systems not being updated in a timely manner), this could also influence the quality-of-care consumers receive.

Similar to other studies, (see [39, 45, 46]), study participants recognised traditional boundaries and hierarchies present in the health care system and attempted to position NPs and PPs within this structure. As new roles open up in the health system, friction between these boundaries could increase, potentially negatively impacting care delivery and leading to reinforcement of current, or indeed more, structural barriers. Examples of such barriers, highlighted in the present paper, include current reporting pathways from secondary care clinicians to primary care advanced practitioners. Given the small numbers of advanced practitioners in New Zealand, participants in the present research likely received care in environments where medical interests govern the redistribution of work. In such an environment, work reallocation from medicine to other health professions may reinforce hierarchies in which doctors retain decision-making power around the division of labour and care provision. Introduction of further advanced practice roles may lead to additional segmented hierarchies even within their own professions [47]. Consequently, more research is required to evaluate how consumer perceptions will change over time as NP and PP roles become more established in different practice settings.

Findings from earlier New Zealand research indicate that advanced practitioners may have yet to reach a critical mass and that there is a general perception of limited strategic direction related to these roles [48, 49]. The New Zealand NP role has recently seen rapidly increasing numbers after years of low registration [50], although numbers remain lower than early government predictions [51]. However, PP numbers are significantly lower than government predictions [52] and both New Zealand Schools of Pharmacy chose not to run requisite training in 2020. Recent changes by New Zealand’s pharmacy regulatory body, the Pharmacy Council, to remove postgraduate prerequisites to undertake PP training have also created additional controversy in the sector, with fear from PPs that this signals a ‘dumbing down’ of a new role [53]. As more nurses and (to a lesser extent) pharmacists accept the mantle of advanced practice, and as general health care practices become more familiar with these roles, consumer views on these providers may change. The impact of legislative and practice changes must be carefully monitored by legislative bodies and education providers, particularly as there may be differences in entry-level clinical skills in an evolving workforce.

Study findings emphasise the importance of establishing a unique identity for new roles; there is a need to remove ambiguity surrounding NP and PP roles. This lack of identity resulted in study participants not understanding roles or scopes, suggesting that in general there may be issues for individuals when they attempt to access health care services targeted to their needs. To manage this issue, consumers should be given the tools to understand how to differentiate the skills and scopes of NPs and PPs from those of other health care professionals.

Although preparation for advanced practitioner roles differs globally (and consequently the generalisability of results to an international audience is not guaranteed), findings from this study emphasise the need to enhance individual ‘health professional literacy’, akin to health literacy. Currently, knowledge of what ‘makes’ an advanced practitioner seldom extends beyond single professional groups; there is a need to extend this knowledge to consumers, encouraging ‘health professional literacy’ alongside health literacy so that consumers can know who is providing them with health services. From this position, the population can make informed choices. Such education initiatives should move beyond framing health professional roles within existing workforce/ workplace structures and instead, focus on framing roles in terms of their value add.

### Research limitations

As with most research studies, this research has limitations because of the sampling approach and the geographic location. For example, although directed to select participants randomly, advanced practitioners selected participants and may have not followed protocols. Alongside a limited sample of potential participants for each recruiting NP/ PP, these issues likely affect how findings can be translated to the other practice contexts. Additional research is required to investigate the perspectives of different consumer populations and consumers who are treated by advanced practitioners practising in specific specialist scopes. As part of such work, it would be pertinent to consider the impact of hierarchies on the quality-of-care and safety of health services consumers receive.

## Conclusions

Consumer knowledge of NP and PP advanced practitioner roles and perception of skills is deficient. Instead, consumers appear to understand NP and PP roles by what they are not, placing them into an established hierarchy. Without complete knowledge about the roles, individuals may fail to engage fully in having services delivered differently within health care settings. As part of using health care resources more effectively, information should be provided to consumers so that they can make informed choices when seeking health care professional support. It is important to review this information as primary health care practices evolve, and as the health system introduces even more models of service delivery. This paper, therefore, contributes to practice by highlighting the need for ‘health professional literacy’ so individuals can make informed choices about accessing services from their health professionals. Ultimately, this research reinforces the importance of ensuring consumers are actively involved in the development of health services, including when advanced practitioner roles are embedded in practice.

## Supplementary Information

**Additional file 1.** Interview schedule

## Data Availability

Restrictions imposed by the Victoria University of Wellington Human Ethics Committee prohibit the provision of complete interview transcripts, as these transcripts contain potentially identifiable and sensitive information. Reflecting this, participant consent forms do not allow the researchers to upload full transcripts.

## Abbreviations

NP: Nurse practitioner
PP: Pharmacist prescriber
GP: General practitioner

## Footnotes

1 The term consumer is used throughout this paper to denote a patient/ service user. We have chosen to use this term as the focus of this paper is around hierarchies and power relationships. Unlike patients/ service users, consumers have agency and should therefore, be empowered to make informed choices around services they receive.
