# Supplementary material for: Perceptions of underlying practice hierarchies: Who is managing my care?
Source: BMC Health Serv Res. 2021 Sep 3;21:911. doi: 10.1186/s12913-021-06931-1 (PMC8414878; doi:10.1186/s12913-021-06931-1)
Supplement: Supplementary file 1 — Additional file 1. Interview schedule [file 12913_2021_6931_MOESM1_ESM.docx]

Interview schedule

| Key questions | Prompts |
| --- | --- |
| What is the role of NPs/PPs in your primary health care practice? | - Can you please provide some examples of their role in your care? |
|  | - What reasons do you have for using NP/PP services over other available care providers? |
| How does their role differ from what you expected? | - Positive factors affecting the consultation process |
|  | - Negative factors affecting the consultation process |
|  | - How do these factors affect consultations? |
| What impact does your NP/PP have on your treatment? | - What do you most value about NP/PP consultations? |
|  | - How does this differ from other consultations you have had? |
|  | - How does the NP/PP change your access to care? |
| Is there anything else that you would like to add? | |
